# Youth Who Sexual Offended: Primary Human Goods and Offense Pathways

**DOI:** 10.1177/1079063213499188

**Published:** 2015-04

**Authors:** Chi Meng Chu, Li Lian Koh, Gerald Zeng, Jennifer Teoh

**Affiliations:** 1 Ministry of Social and Family Development, Singapore; 2 Monash University, Melbourne, Australia

**Keywords:** child victim, good lives model, offense pathways, penetrative sexual offenses, primary human goods, self-regulation model

## Abstract

There has been an increased focus on understanding youth sexual offending in recent years, but there has been limited empirical research on the causes, pathways, and treatment of youth who have sexually offended—especially within a non-Western context. The Good Lives and Self-Regulation Models have often been used to understand and rehabilitate adult sexual offenders, but (unfortunately) there is scant research on youth who sexually offended using these models. The present study aims to describe the different primary goods that are associated with youth sexual offending behaviors in an Asian context. In addition, the study sought to explore whether the age of victim (child vs. nonchild) and nature of sexual offense (penetrative vs. nonpenetrative) influenced the youth’s engagement in offense pathways. The results suggest that pleasure, relatedness, and inner peace were the primary human goods that were most sought after by a sample of 168 youth who sexually offended in Singapore. In addition, offender classification (in relation to the age of victim and nature of sexual offense) influenced the pathways to sexual offending. Therefore, these findings have important clinical implications for assessment, management, and intervention planning for youth who sexually offended.

## Introduction

Early work on understanding sexual offending has been mostly focused on adult offenders, but youth sexual offending represents an ongoing social, judicial, clinical, and policy issue for services. Although there is a greater focus on understanding youth sexual offending in recent years (see Seto & Lalumière, 2010, for a review), there is a relative paucity of empirical research on the causes, pathways, and treatment of youth who sexually offended especially within a non-Western context.

### Good Lives Model (GLM)

The GLM is a contemporary, strength-based approach to offender rehabilitation that emphasizes the promotion of individuals’ personal goals, concomitant with the reduction or management of their risk of future offending (Laws & Ward, 2011; Ward, 2002; Ward & Maruna, 2007; Ward & Stewart, 2003). According to the GLM, primary human goods are characteristics, experiences, and/or states of mind that are valued by individuals, and will increase the individuals’ sense of fulfillment and happiness (Ward, 2002). Some examples of primary human goods include (a) community (connection to wider social groups), (b) creativity (expressing oneself through alternative forms), (c) excellence in agency (autonomy and self-directedness), (d) excellence in play (hobbies and recreational pursuits) and work (mastery experiences), (e) inner peace (freedom from emotional turmoil and stress), (f) knowledge (how well informed one feels about things that are important to them), (g) life (healthy living and functioning), (h) happiness/pleasure (the state of feeling good in the here and now), (i) relatedness (peer, romantic, and familial relationships), and (j) spirituality (in the broad sense of finding purpose and meaning in life; Ward & Gannon, 2006).

However, secondary goods represent concrete means or activities that are undertaken in pursuit of the primary human goods. For example, knowledge might be achieved through attending university classes or workshops. It is assumed that all individuals seek these goods, albeit to different degrees according to their values, developmental stage, and priorities in life. The attainment of these goods is usually associated with higher levels of well-being, as well as the development of a self-identity and purpose in life, whereas the converse is associated with psychological problems (e.g., Emmons, 1999; Ward & Stewart, 2003; Yates & Ward, 2008).

Offending behaviors are viewed as flawed attempts at gaining fulfillment in the individuals’ lives and could result from (a) engagement in inappropriate or harmful means to obtain the primary human goods, (b) pursuit of an overly narrow range of primary human goods at the expense of other more important ones, (c) a lack of coherence between the valued primary human goods and the means used to obtain them, and/or (d) a lack of necessary competencies, opportunities, and/or resources to obtain the primary human goods (Ward, Yates, & Willis, 2012). Within the GLM framework, the offenders’ personal preferences and values, as well as the significance of their primary human goals, are acknowledged and used to motivate these offenders to live better lives. Importantly, the therapists are encouraged to assist the offenders to obtain the necessary competencies and opportunities to work on treatment plans likely to ultimately help them fulfill their primary human goods.

### Empirical Research on the GLM

Ward and Mann (2004) and Yates, Prescott, and Ward (2010) suggested that the inability to attain primary human goods such as excellence in agency, inner peace, happiness (which includes pleasure), and relatedness is more strongly associated with sexual offending because these goods are closely linked to dynamic risk factors for sexual offending. Barnett and Wood (2008) conducted a study with adult sexual offenders to examine the differences in priority that was assigned to the primary human goods of agency, inner peace, and relatedness at the time of sexual offending. The results showed that adult sexual offenders had prioritized agency and relatedness ahead of inner peace; however, it should noted that the sample was small (*N* = 42) and the other primary goods were not examined in the study. In another study of primary human goods in an adult sexual offender sample (*N* = 96), Yates, Kingston, Simons, & Tyler (2009) found that agency (36.5%), inner peace (25.0%), happiness (19.8%), and relatedness (14.6%) were the most-valued goods, and that the offenders’ prioritization of happiness above all other primary goods was associated with the number of victims and with paraphilia.

### Self-Regulation Model (SRM)

Through a grounded theory approach, Ward and Hudson (1998) had developed the SRM as an alternative to relapse prevention. The SRM posits that individuals are goal-directed in their behavior, whereby they act to either achieve a desired state or to avoid an undesired state (Yates et al., 2010). The SRM also describes different routes to offending in which offenders can be categorized, and these pathways are defined by the offender’s goal toward sexual offending (approach vs. avoidant) and the manner in which the offender attempts to attain this goal (passive vs. active).

Approach goals pertain to the successful achievement of a particular state or situation, whereas avoidant goals, on the other hand, seek to reduce or minimize a particular state or situation (Cochran & Tesser, 1996). Offenders with passive self-regulation are postulated to be underregulated in their behavior and might typically respond to situations and emotional cues in ways intended to modulate or heighten arousal (Ward & Hudson, 1998). Offenders with an active self-regulatory style typically engage in goal-directed behavior (e.g., grooming victims) or selecting inappropriate strategies to self-regulate. As such, four different pathways of offending are proposed by the SRM: (a) Avoidant-Passive (i.e., the offender wants to avoid sexual offending but lacks the necessary skills to prevent it from happening); (b) Avoidant-Active (i.e., the offender directly tries to control deviant thoughts and fantasies but uses ineffective or counterproductive strategies); (c) Approach-Automatic (i.e., the offender has overlearned sexual scripts for offending and engages in poorly planned behavior); and (d) Approach-Explicit (i.e., the offender desires to sexually offend, harbors harmful goals pertaining to sexual offending, and plans to execute offenses; Yates et al., 2010; Yates & Ward, 2008).

### Empirical Research on the SRM

Table 1 summarizes the findings of several empirical studies that have been conducted on adult sexual offenders and the SRM. Taken together, the studies suggest that Avoidant pathway offenders tend to have sexual interest in children and are also likely to engage in intrafamilial offending (see Bickley & Beech, 2002; Kingston, Yates, & Firestone, 2012; Kingston, Yates, Simons, & Tyler, 2009; Yates & Kingston, 2006); whereas rapists and those who sexually offended against male children tended to follow the Approach-Explicit pathway. For sexual offenders with special needs and intellectual disability, the extant literature indicates that they are likely to follow the Avoidant pathways (see Ford, Rose, & Thrift, 2009; Keeling, Rose, & Beech, 2006; Langdon, Maxted, Murphy, & the Sex Offenders Treatment Services Collaborative-Intellectual Disabilities Group, 2007). However, it is noted that these studies are somewhat weak methodologically. Overall, there is some evidence to suggest that different types of adult sexual offenders are associated with different offense pathways.

**Table 1.** Empirical Studies on Adult Sexual Offenders and the Self-Regulation Model.

Study	Summary of relevant findings
Bickley and Beech (2002; *N* = 87)	80% had approach goals and >50% used active strategies to achieve these. Few followed the Av-P pathway. Offenders following different offense pathways could be distinguished by characteristics (e.g., cognitive distortions, emotional congruence with children, victim types, and previous convictions).
Webster (2005; *N* = 25)	Ap-E pathway was predominant for this sample before and after treatment. There was no support for the notion that offenders would change pathways following treatment.
Yates and Kingston (2006; *N* = 80)	Av-P and Ap-A pathways were associated predominantly with incest offenders and rapists, respectively. For the Ap-E pathway, it comprised nearly equal numbers of rapists, child molesters against male victims, and incest offenders. The pathways were differentially associated with offender types.
Keeling, Rose, and Beech (2006; *N* = 64)	Special needs offenders were compared with mainstream offenders. All but one offender had approach goals and almost 2/3 had a passive self-regulation style. Ap-E and Ap-A pathways were most common for mainstream offenders, and the latter for special needs offenders.
Langdon, Maxted, Murphy, and the Sex Offenders Treatment Services Collaborative-Intellectual Disabilities Group (2007; *N* = 34)	Offenders with intellectual disability followed predominantly Ap-A and Ap-E offense pathways. Approach pathway offenders had higher levels of cognitive distortion and more denial about the negative impact of their offending on victims. No differences between offense pathways in terms of victim types.
Ford, Rose, and Thrift (2009; *N* = 28)	Offenders with intellectual disability followed predominantly Ap-A and Ap-E offense pathways. Offenders with passive self-regulation had lower intellectual functioning than those with active self-regulation.
Kingston, Yates, Simons, and Tyler (2009; *N* = 96)	Offenders with Ap-E pathway were most likely to offend against children. However, Ap-E and Ap-A pathway offenders were more likely than Avoidant pathway offenders to show sexual interest in children. Ap-A offenders were most likely to use interpersonal violence, but Av-P and Av-A pathway offenders were least likely.
Kingston, Yates, and Firestone (2012;*N* = 275)	Rapists predominantly followed the Ap-A pathway, extrafamilial child molesters and mixed offenders followed the Ap-E pathway, and intrafamilial child molesters followed the Av-P pathway. Multivariate analyses revealed that Approach pathway offenders exhibited more problematic offense characteristics as well as higher risk and treatment need than those with inhibitory goals.

*Note.* Av-A = Avoidant-Active; Av-P = Avoidant-Passive; Ap-E = Approach-Explicit; Ap-A = Approach-Automatic.

### Combining the GLM and SRM

Yates et al. (2010) have recently demonstrated how the SRM can be integrated within the overarching framework of the GLM, effectively targeting offenders’ risk while also enhancing their levels of well-being. The integration of the GLM and SRM allows the evaluation of the individual’s good lives plan, its scope, individuals’ internal capacity to obtain primary human goods and to regulate behavior, as well as the external opportunities that facilitate or constrain the implementation of good lives plans. In addition, it enables the assessment of self-regulation capacity and the offense pathways (Yates & Ward, 2008). Taken together, the integrated model has important implications for the management and treatment of sexual offenders.

### Youth Who Sexually Offended Against Child Victims

Several typologies of youth who sexually offended have been postulated in the extant literature, and one of these typologies concerns youth who sexually offended against child victims^1^ (see Fanniff & Kolko, 2012, for a review). Scholars have suggested that youth who sexually offended are different in terms of their sexual interests toward children and coercive sex with peers or adults when compared with youth who sexually offended against nonchild victims, and these atypical sexual interests may motivate their sexual offenses (e.g., Becker & Kaplan, 1988; Finkelhor, 1984; Hall, & Hirschman, 1991; Marshall & Barbaree, 1990; Seto, Murphy, Page, & Ennis, 2003; Ward & Siegert, 2002). Specifically, Seto at al. (2003) found, in a sample of youth who sexually offended, that sexual arousal to stimuli depicting children was associated with child victim preferences. Moreover, studies on youth who sexually offended have shown that sexual arousal to stimuli depicting children predicted sexual recidivism (Clift, Rajlic, & Gretton, 2009; Rice, Harris, Lang, & Chaplin, 2012), and that self-reported sexual interest in children was also associated with sexual recidivism (Worling & Curwen, 2000). These findings were generally similar to those found in adult sexual offenders (Hanson & Morton-Bourgon, 2005).

Compared with youth who sexually offended against nonchild victims, individuals who sexually offended against child victims were younger at age of offense, more likely to be familiar with the victims, less likely to use force in their offenses, had more severe sexual offenses, were more deficient in social functioning, more likely to have psychosexual development difficulties, and more likely to have depressive and anxiety symptoms (e.g., Aebi, Vogt, Plattner, Steinhausen, & Bessler, 2012; Gunby & Woodhams, 2010; Hart-Kerkhoffs, Doreleijers, Jansen, van Wijk, & Bullens, 2009; Hendriks & Bijleveld, 2004). Despite these abovementioned findings, there is a lack of studies that have examined the primary goods and offense pathways in youth who sexually offended against child victims.

### Youth Who Sexually Offended in Singapore

Singapore is an independent island-state in South East Asia and is a member of the Commonwealth of Nations. Many statutes are based on English common law (e.g., the Criminal Procedure Code, 2012). However, there are some statutes that are based on legislation from other jurisdictions; for example, the Penal Code (2008) is based on the Indian Penal Code, which was (nonetheless) first formulated by the English in 1800s. As such, there are many similarities in the way that sexual offenses are defined in Singapore when compared with the abovementioned countries, even if the exact language of the laws might differ somewhat.

Sexual offenses account for about 4% to 5% of all crimes in Singapore with most recorded as molestation offenses (i.e., groping and nonpenetrative touching; Singapore Police Force, 2012). Using criminal records data, Chu and Thomas (2010) found that 11.5% of a Singaporean sample of youth who sexually offended had sexually reoffended, and differed from their Western counterparts in some characteristics. For example, research from Western countries showed that youth who sexual offended were unlikely to sexually assault strangers (e.g., Vizard, Hickey, French, & McCrory, 2007), whereas the majority of the Singaporean sample had sexually offended against strangers (Chu & Thomas, 2010). In addition, Chu and Thomas revealed that youth who sexually offended in Singapore were less likely to have offended against male victims as compared with published Western findings (e.g., Kemper & Kistner, 2007; Nisbet, Wilson, & Smallbone, 2004; Vizard et al., 2007).

Cultures and societies often define what kind of attitudes and behaviors are considered “normal” and “deviant.” Notwithstanding that there is some degree of consensus across cultures about what constitutes sexually deviant attitudes and behaviors (e.g., molestation, rape, and paraphilia), development of sexually deviant attitudes and behaviors can vary due to cultural norms, gender roles, morals, religion, taboos, and expectations (see Bhugra, Popelyuk, & McMullen, 2010, for a review). Moreover, there could also be cross-cultural differences as to how individuals cope, self-regulate, or even report sexual crimes. Therefore, it is possible that the motivation, risk factors, and pathways for sexual offending may differ cross-culturally. Currently, the primary human goods that are associated with the youth’s sexual offending, as well as their offense pathways, have not been examined in the Asian or Singaporean context.

### Aims of the Present Study

Despite the promise of the GLM and SRM for sexual offender rehabilitation (see Willis, Yates, Gannon, & Ward, 2013; Yates & Ward, 2008), there has been a dearth of empirical studies to elucidate the primary human goods that are linked to offense pathways of youth who sexually offended. As such, the present study aims to explore whether youth who had committed sexual offenses sought different primary human goods that are linked to their sexual offending behavior within an Asian context, and also engaged in different offense pathways as defined by Ward and Hudson (1998). The present study represents a preliminary investigation of the degree to which primary human goods and the four self-regulation pathways are evident in file accounts relating to the offending behavior of youth who sexually offended in Singapore.

## Method

### Source Sample

The sample consisted of 168 male youth who sexually offended (aged 12-18 years). They were referred to the Clinical and Forensic Psychology Branch (CFPB) of the Ministry of Social and Family Development (Singapore) between October 2002 and March 2012 for a psychological assessment of their risk of future sexual offending after they were charged and convicted of sexual offenses. The current sample included *all* the youth who sexually offended who were referred to CFPB during this period^2^ (see Table 2 for sample characteristics).

**Table 2.** Characteristics for the Youth Who Sexually Offended.

Variables	*M*	*SD*	Range
Age at referral	14.92	1.43	12-18
Number of current offenses	4.76	5.13	1-39
Number of current sexual offenses	3.82	4.71	1-39
ERASOR total score	36.19	6.20	17-52
	*n*	%	
Source of referral			
Probation services	120/168	71.4	
Youth correctional institutions	34/168	20.2	
Child protection services	12/168	7.4	
Ethnicity of youth			
Chinese	75/168	44.6	
Malay	68/168	40.5	
Indian	20/168	11.9	
Other	5/168	3.0	
Intellectually disabled	20/168	11.9	
Nature of sexual offense			
Nonpenetrative sexual offense	119/168	70.8	
Penetrative sexual offense	49/168	29.2	
Age of victim(s)			
Child	45/168	26.8	
Nonchild	123/168	73.2	
Offender classification			
Nonchild-nonpenetrative	90/168	53.6	
Nonchild-penetrative	33/168	19.6	
Child-nonpenetrative	29/168	17.3	
Child-penetrative	16/168	9.5	
Criminally diverse^a^	56/168	33.3	
Also committed violent offense(s)^b^	18/168	10.7	
Also committed nonviolent nonsexual offense(s)^c^	38/168	22.6	

a Criminally diverse refers to committing nonsexual offenses in addition to sexual offenses.

b For example, causing bodily harm, rioting, and robbery.

c For example, burglary, drug use, fraud, and theft.

### Ethical Approval

Approval for the research study was obtained from the CFPB of the Ministry of Social and Family Development before the commencement of the study.

### Offender Classification

The youth who sexually offended in this study were classified according to two factors: (a) the age of victim and (b) the nature of sexual offense.

#### Age of victim

In this study, a “child victim” was defined as a victim who is under the age of 12 years and at least 4 years younger than the youth who sexually offended, whereas a victim who did not meet these criteria was classified as a “nonchild victim.” Information from the charge sheets and statement of facts were used for this classification. This definition is consistent with that adopted in a well-validated youth risk assessment measure, the Estimate of Risk of Adolescent Sexual Offense Recidivism (ERASOR; Worling & Curwen, 2001). For the purpose of this study, those who committed sexual offenses against child and nonchild victims were classified as youth who have assaulted child victims.

#### Nature of sexual offense

The youth were further classified into two groups: those who had committed (a) penetrative sexual offenses and (b) nonpenetrative sexual offenses. For the purpose of this study, those youth who had committed penetrative and nonpenetrative sexual offenses were classified as “youth who committed penetrative sexual offenses” given that the former is more intrusive and viewed as more serious. It was of interest to examine if there were differences between these two groups especially with regard to the offense pathways given that severity of offenses is an important consideration in risk assessment and management. Penetrative sexual offenses included sodomy, nonconsensual oral sex, rape, as well as digital penetration. Nonpenetrative sexual offenses included molestation (e.g., groping and nonpenetrative touching), voyeuristic (e.g., peeping at others changing or taking upskirt photographs), exhibitionistic (e.g., exposing oneself to nonconsensual others or in public places), and any other sexual offenses that did not involve physical contact with victims. Charge sheets and statement of facts that were available in the file records were used to assist with the classification (see Table 2 for a breakdown of the offender classification).

### Measures

The ERASOR and the Offense Pathways Checklist were used in this study. The ERASOR is a validated risk assessment measure, whereas the Offense Pathways Checklist is a simple clinical protocol that provides rating guidelines on the offense pathways.

#### ERASOR

The ERASOR (Worling & Curwen, 2001) is an empirically guided, structured clinical judgment measure that comprises 25 items (16 dynamic and 9 static risk factors) and is designed to assist clinicians in estimating the risk of sexual recidivism for youth (aged 12-18 years) who have sexually offended. The items are grouped into five sections: Sexual Interests, Attitudes, and Behaviors; Historical Sexual Assaults; Psychosocial Functioning; Family/Environmental Functioning; and Treatment. Each item can be coded as *unknown* (0), *not present* (1), *possibly/partially present* (2), or *present* (3). We obtained a total score from summing the item scores; this was used as a covariate in our multivariate analyses. For the purpose of this study, Items 24 and 25 from Treatment section of the ERASOR (i.e., *No development or practice of realistic plans/strategies*, and *Incomplete sexual-offense-specific treatment*) were not scored as information obtained during the psychological assessment stage were only used for rating of the ERASOR—information pertaining to treatment were not used for the scoring (please see the “Procedure” subsection). The ERASOR has been shown to have good reliability (e.g., intraclass correlation coefficients [ICCs] > .80 for total score and clinical judgment rating; Worling, Bookalam, & Litteljohn, 2012), and moderate predictive validity for predicting sexual recidivism (e.g., weighted Area Under Curve = .66 for total score and clinical judgment rating; Viljoen, Mordell, & Beneteau, 2012). Moreover, it has been validated in the Singaporean context (Chu, Ng, Fong, & Teoh, 2012).

#### Offense Pathway Checklist

This self-regulation rating protocol consists of nine items that are rated on a scale of 0 to 10. Of these nine items, five pertained to the Passive/Active dimension (i.e., *Degree of Planning; Degree of Thought Before Acting; Complexity of Strategies Used [Either to Offend or Prevent Offending]; Locus of Control [Victim Stance]*; and *Ability to Delay Gratification*), whereas another four pertained to the Avoidant/Approach dimension (i.e., *Reported Desire to Control/Prevent Offending; Beliefs about Children and Sex [Cognitive Distortions]; Degree of Guilt/Shame Following Offense*; and *Level of Pro-Offending Behaviors*). The scores for the dimension items were summed to obtain a Total Score for the Avoidant/Approach dimension (total score = 0-40) and Passive/Active dimension (total score = 0-50). Several studies have utilized this checklist (e.g., Bickley & Beech, 2002; Ford et al., 2009; Keeling et al., 2006; Langdon et al., 2007) and the interrater reliability was found to be moderate to excellent (kappa = .60-.83; see Cicchetti, 1994, for a classification of kappa).

### Procedure

The current study was retrospective in nature. For the purpose of this study, two psychologists from CFPB (the second and third authors), who were trained in conducting risk assessments for youth who sexually offended, had conducted clinical file reviews. They had completed coding for the primary human goods as well as the pathways based on file information. The clinical files contained (a) reports prepared by CFPB psychologists, (b) presentencing reports prepared by probation officers, (c) institution risk and needs reports, (d) charge sheets, (e) statement of facts, (f) any previous assessment and treatment reports on the youths’ CFPB files, and (g) school reports.

As the psychological interviews conducted at the CFPB follow a standardized semistructured interview schedule, the resultant psychological reports contain specific information pertaining to several key areas of assessment (i.e., personal, family, psychiatric, and criminal offending histories as well as the current offending behaviors and risk management issues). These areas yielded valuable information about the youth’s upbringing, interaction with peers and authorities, general and academic functioning, values, family and school environments, as well as information relating to his offending. The statement of facts and charge sheets, presentencing report, as well as psychological reports contain extensive information on the sexual offenses. Furthermore, the psychological and probation officers’ reports typically contain useful information that pertained to the types of primary human goods that are implicated in their sexual offending behavior.

Pertaining to the primary human goods, the psychological reports and file records were used to determine whether there was evidence (i.e., *Present* or *Absent*) that any of the 10 primary human goods (i.e., community, creativity, excellence in agency, excellence in play and work, inner peace, knowledge, life, pleasure, relatedness, and spirituality) were related to their sexual offending (at the point of initial assessment). The definitions of these 10 primary human goods were adopted from the descriptions provided in Ward and Gannon (2006).

For training purposes, the second and third authors had attended GLM and SRM training workshops (conducted by Tony Ward and colleagues), and had operationalized the coding process beforehand. The raters then coded five cases, and further discussed any discrepancies in detail to ensure a high level of consistency in their coding. To examine the interrater reliability for ratings pertaining to the primary human goods, offense pathways, and the ERASOR, they had separately coded a randomly selected sample of 16 (9.82%). The intraclass correlation coefficients for single rater (using absolute agreement definition; ICCs) were 1.00 (*excellent*) for all the primary goods (except inner peace, which was .78 [*excellent*]), .91 (*excellent*) for the Passive/Active dimension total score, .78 (*excellent*) for the Avoidant/Approach dimension total score, and .69 (*good*) for the ERASOR total score (see Cicchetti, 1994, for a classification of ICCs).

### Statistical Analyses

The sample was characterized using descriptive statistics, with categorical data reported as frequencies and percentages, and continuous data presented in relation to the mean and standard deviation. The continuous data were plotted to check for skewed distributions. Chi-square tests of association were computed for categorical data, and effect sizes were computed to demonstrate the strength of the associations between variables. A one-way ANOVA was conducted to test for differences between the offender classifications in terms of ERASOR total scores. In addition, a scatter plot was used to check the distribution of the youth who had (a) targeted child and nonchild victims, and (b) committed penetrative and nonpenetrative sexual offenses on the scales for Avoidant/Approach and Passive/Active dimensions in terms of the offense pathways. Two-way MANCOVA and one-way ANOVA were conducted to test differences in the dimension scale scores for the factors relating to the age of the victim (i.e., child and nonchild victims) and the nature of the sexual offense (i.e., penetrative vs. nonpenetrative), and to examine any interaction effect between aforementioned factors. The total risk score from the ERASOR was entered as a covariate in the MANCOVA to control for differences in the risk of sexual recidivism of the youth. Analyses were conducted using SPSS version 19.

## Results

### ERASOR Ratings and Offender Classifications

The offender classifications were found to differ significantly in terms of ERASOR total score, *F*(3, 164) = 13.81, *p* ≤ .001, $\eta_{p}^{2}$ = .20. Post hoc tests (Tukey HSD) revealed that the mean ERASOR total scores differed significantly between the following classifications: (a) nonchild-nonpenetrative (*M* = 34.03, *SD* = 5.49) and child-nonpenetrative (*M* = 41.31, *SD* = 5.61), (b) nonchild-nonpenetrative (*M* = 34.03, *SD* = 5.49) and child-penetrative (*M* = 39.00, *SD* = 6.24), as well as (c) nonchild-penetrative (*M* = 35.91, *SD* = 5.66) and child-nonpenetrative (*M* = 41.31, *SD* = 5.61).

### Primary Human Goods Sought by Youth Who Sexually Offended

Table 3 shows the primary human goods that were implicated in the youth’s sexual offending. A vast majority of the youth who sexually offended had sought pleasure (91.1%; 153/168). The next most sought-after primary human goods that were implicated in their sexual offending were relatedness (35.7%; 60/168) and inner peace (17.3%; 29/168). Table 3 also shows the primary human goods that were sought by youth who committed sexual offenses against child and nonchild victims, as well as penetrative and nonpenetrative offenses. Chi-square tests indicated that the associations between the classifications (child vs. nonchild victim, and penetrative vs. nonpenetrative) and the primary human goods were nonsignificant.

**Table 3.** The Primary Human Goods That Were Sought by Youth Who Committed Sexual Offenses Against Child and Nonchild Victims, as Well as Penetrative and Nonpenetrative Offenses.

		Age of victim		Nature of offense	
Primary human goods sought	Total sample (*N* = 168)	Child victim (*n* = 45)	Nonchild victim (*n* = 123)	Penetrative (*n* = 49)	Nonpenetrative (*n* = 119)
Community	15/168 (8.9%)	3/45 (6.7%)	12/123 (9.8%)	3/49 (6.1%)	12/119 (10.1%)
Creativity	0/168 (0%)	0/45 (0%)	0/123 (0%)	0/49 (0%)	0/119 (0%)
Excellence in agency	9/168 (5.4%)	4/45 (8.9%)	5/123 (4.1%)	1/49 (2.0%)	8/119 (6.7%)
Excellence in play	4/168 (2.4%)	1/45 (2.2%)	3/123 (2.4%)	0/49 (0%)	4/119 (3.4%)
Excellence in work	6/168 (3.6%)	2/45 (4.4%)	4/123 (3.3%)	2/49 (4.1%)	4/119 (3.4%)
Inner peace	29/168 (17.3%)	9/45 (20.0%)	20/123 (16.3%)	5/49 (10.2%)	24/119 (20.2%)
Knowledge	3/168 (1.8%)	1/45 (2.2%)	2/123 (1.6%)	0/49 (0%)	3/119 (2.5%)
Life	0/168 (0%)	0/45 (0%)	0/123 (0%)	0/49 (0%)	0/119 (0%)
Pleasure	153/168 (91.1%)	42/45 (93.3%)	111/123 (90.2%)	48/49 (98.0%)	105/119 (88.2%)
Relatedness	60/168 (35.7%)	14/45 (31.1%)	46/120 (37.4%)	13/49 (26.5%)	47/119 (39.5%)
Spirituality	0/168 (0%)	0/45 (0%)	0/123 (0%)	0/49 (0%)	0/119 (0%)

*Note.* Chi-square analyses revealed that all of the comparisons for (a) child versus nonchild victim, and (b) penetrative versus nonpenetrative offenses were nonsignificant at *p* = .05.

### Offender Classification and Offense Pathways

**Figure 1.**
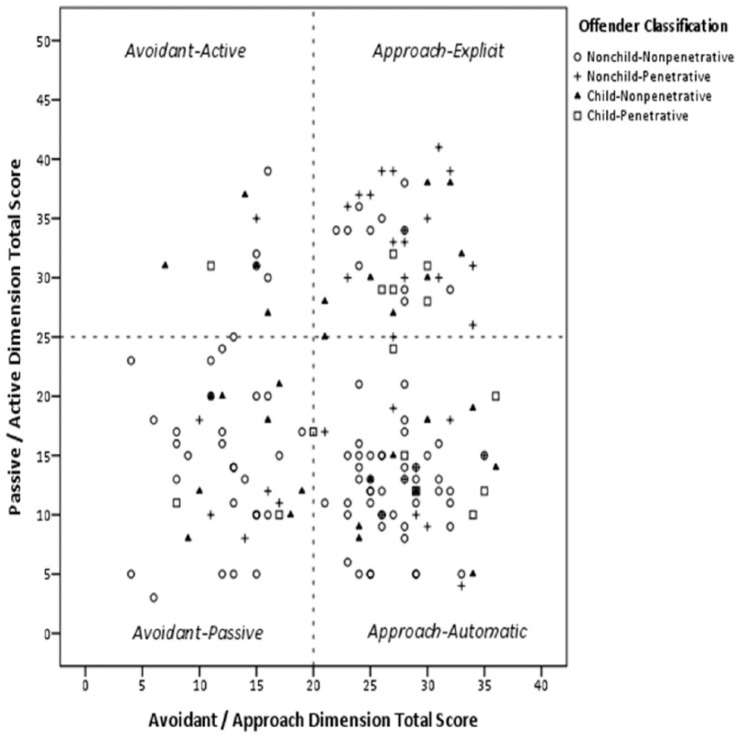
Scatter plot of offender classification and total scores for offense pathway dimensions.

The youth who sexually offended against child victims were not more likely to commit penetrative offenses (35.6%, 16/45) than youth who sexually offended against nonchild victims (26.8%, 33/123), χ^2^(1) = 1.21, *ns*. Figure 1 shows the scatter plot of the offender classification and total scores for the offense pathway dimensions. More than a third of the sample (43.9%, 72/164^3^) were found in the Approach-Automatic quadrant of the scatter plot, and another quarter (26.8%, 44/164) were in the Avoidant-Passive quadrant. The two most prevalent pathways for the offender classifications were (a) nonchild-nonpenetrative: Approach-Automatic (50.6%, 45/89) and Avoidant-Passive (32.6%, 29/89); (b) nonchild-penetrative: Approach-Explicit (50%, 16/32) and Approach-Automatic (31.3%, 10/32); (c) child-nonpenetrative: Approach-Automatic (32.1%, 9/28) and Avoidant-Passive (32.1%, 9/28); and (d) child-penetrative: Approach-Automatic (53.3%, 8/15) and Approach-Explicit (33.3%, 5/15).

**Figure 2.**
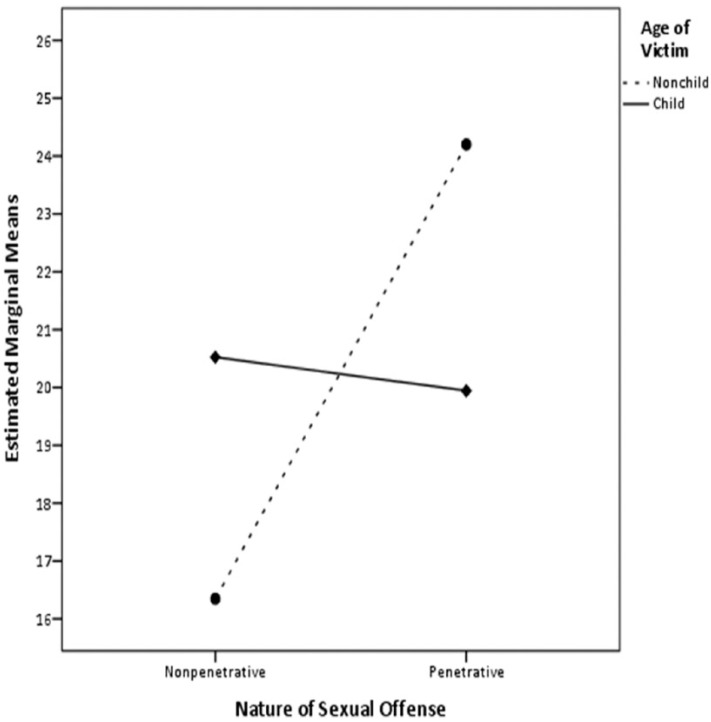
Interaction effect between age of victim and nature of sexual offense on Passive/Active dimension total scores after controlling for ERASOR total scores. *Note.* ERASOR = Estimate of Risk of Adolescent Sexual Offense Recidivism.

A two-way MANCOVA was undertaken to explore the effect of the age of victim and the nature of sexual offense on offense pathways, controlling for the differences in ERASOR total score. The variance−covariance matrices were found to be homogeneous (i.e., Box’s Test was nonsignificant), and hence the Wilks’s λ was used. The nature of sexual offense (i.e., penetrative vs. nonpenetrative) influenced the offense pathways (i.e., Avoidant/Approach and Passive/Active), *F*(2, 162) = 6.66, *p* = .002,$\eta_{p}^{2}$ = .08 after controlling for risk of sexual recidivism. In addition, an interaction between the nature of sexual offense and age of victim (i.e., child vs. nonchild) tended to influence the offense pathways, *F*(2, 162) = 2.90, *p* = .058, $\eta_{p}^{2}$ = .04. Univariate one-way ANOVAs revealed that the nature of sexual offense did indeed influence Passive/Active dimension, *F*(1, 163) = 4.08, *p* = .045, $\eta_{p}^{2}$ = .02, and Avoidant/Approach dimension, *F*(1, 163) = 9.53, *p* = .002, $\eta_{p}^{2}$ = .06. Furthermore, the interaction between age of victim and nature of sexual offense influenced the Passive/Active dimension of the offense pathways, *F*(1, 163) = 5.36, *p* = .022, $\eta_{p}^{2}$ = .03 (see Figure 2). Table 4 presents the estimated marginal means and standard errors of the Passive/Active and Avoidant/Approach dimension total scores as a function of offender classification.

**Table 4.** The Estimated Marginal Means and Standard Errors of the Passive/Active and Avoidance/Approach Dimension Total Scores as a Function of Offender Classification.

	Passive/Active score		Avoidance/Approach score	
Offender classification	*M*	*SE*	*M*	*SE*
Nonchild-nonpenetrative (*n* = 90)	16.35	1.06	22.74	.77
Nonchild-penetrative (*n* = 33)	24.20	1.69	25.92	1.23
Child-nonpenetrative (*n* = 29)	20.52	1.93	19.30	1.41
Child-penetrative (*n* = 16)	19.94	2.45	24.23	1.79

## Discussion

### Comparisons With Extant Literature

It appeared that the pleasure (91.1%) was the most sought good in relation to the youth’s sexual offending regardless of their classification. Relatedness (35.7%) and inner peace (17.3%) were the next most-sought-after goods. The findings are not surprising given that adolescence is often seen as a period of growth, development, and change (Arnett, 1999). In particular, youth have been reported to be high in sensation seeking and strongly motivated to establish relationships with their peers (Eaton et al., 2008; Erikson, 1968; Steinberg et al., 2008), but some may lack the appropriate means to do so. Furthermore, they may have erroneously chosen to inoculate or free themselves from emotional turmoil and stress through sexual offending. Although the differences were nonsignificant across the classification variables (i.e., age of victim and nature of sexual offense), the primary human goods (i.e., pleasure, relatedness, and inner peace) linked to the youth’s sexual offending behaviors are generally consistent with their developmental stage and suggest that fulfillment of these goals are key tasks during this stage of their lives.

With regard to the SRM, the results from the present study provided support to the conclusion that, similar to adult sexual offenders, multiple pathways exist for youth sexual offending rather than a single route (Kingston et al., 2012; Yates & Kingston, 2006). Importantly, these pathways may differ as a result of offender classification, and our results suggest that these offense pathways also apply within an Asian context. Consistent with Bickley and Beech’s (2002) and Keeling et al.’s (2006) findings, the majority of the youth who sexually offended followed the Approach pathways. Closer examination of the offender classifications revealed that youth who committed penetrative offenses tended to follow the Approach pathways, whereas those who molested children tended to follow the Avoidant-Passive and the Approach-Automatic pathways; these findings were somewhat consistent with the literature about the offense pathways for adult rapists and child molesters (Kingston et al., 2012; Yates & Kingston, 2006).

Considering that adolescence is often associated with sensation seeking, impulsivity, and stress relief/inoculation, it is thus unsurprising that a substantial proportion of the present sample follow the Approach- and Passive-oriented pathways—the combination that Kingston et al. described as being associated with offense progression and suggestive of impulsivity and general criminality in adult sexual offenders. Other explanations for youth sexual offending (e.g., social incompetence, atypical sexual interests, poor sexual development, etc.) as well as the aforementioned developmental aspect should be further examined in future studies on the SRM. Given that individuals with Asian backgrounds generally use more passive and emotion-focused coping strategies than their Western counterparts (see Kuo, 2011, for a review), a key area to examine is whether youth across cultures have different regulation strategies for their deviant sexual interests and associated affective states. Comparative studies conducted in Western and non-Western contexts (which is lacking at the moment) will be integral for distinguishing the cultural contributions.

There were significant differences in the way the youth attempted to attain their goals (i.e., the Passive/Active dimension of the offense pathways) when we examined the victims (child vs. nonchild) they had chosen and the nature of offenses (penetrative vs. nonpenetrative) they had engaged in. Youth who sexually offended against nonchild victims tended to be more passive-oriented than those who offended against child victims in terms of committing nonpenetrative offenses, but this relationship was reversed when it pertained to committing penetrative offenses (see Figure 2). These results suggest that those youth who committed penetrative or nonpenetrative offenses against child victims as well as nonpenetrative offenses against nonchild victims maybe disinhibited in their behavior and may respond in ways that serve to modulate or heighten their arousal (i.e., passive-oriented pathways). In contrast, youth who engaged in penetrative sexual offenses against nonchild victims exhibited greater intentionality with respect to their sexual offending.

Taken together, the range of goods sought and the motivation to pursue them are hypothesized to be innate, and therefore, universal (Ward & Gannon, 2006). The existence of *natural* desires to seek goods such as intimacy, agency, emotional balance, and mastery has been noted in a diverse range of research literature including evolutionary psychology, biology, and personality theory. Importantly, the *content* of beliefs and desires (and values), while tracking common motivational states, are fleshed out by social learning and cultural knowledge in particular contexts. Thus, what actually constitutes good or normal relationships will vary from culture to culture, while the need to establish such relationships is universal. In our view, some of the differences in the behavioral strategies and associated goals evident in our sample most likely reflects cultural differences. This possibility, however, requires greater research attention than we were able to give it in our study.

### Implications

According to Ward and Gannon (2006), sexual offending is likely to reflect the influence of a plethora of goals and the related primary human goods. More specifically, sexual offending is viewed as a manifestation of problematic attempts at seeking the human goods and ultimately fulfillment in the lives of offenders. It may be useful to assess them to enable the clinicians and the youth to appreciate the role of these primary human goods in the youth’s sexual offending as well as the relationship between deficits and their offense-related goals. It is also easier to persuade the youth to abandon criminal lifestyles if they can see that their core commitments have been acknowledged and factored into a rehabilitation plan. This might help to reduce their risk of further offending and consolidating new and fulfilling ways of living. Nevertheless, it should be highlighted that this is a preliminary investigation into the primary human goods that are linked to the youth’s sexual offending. Further research and replication will be needed in this area of work to inform how the clinicians can structure treatment and intervention strategies that will equip the youth with internal resources and external conditions, so that they can achieve these goods in prosocial ways.

With regard to the SRM, it contains a number of pathways, representing different combinations of offense-related goals, and the use of distinct regulation styles in relation to sexually offensive contact (i.e., underregulation, misregulation, and effective regulation; Yates et al., 2010; Yates & Ward, 2008). Information about these offense pathways is important and these should be carefully assessed and subsequently managed. Moreover, the integration of the SRM within the overarching framework of the GLM can target the offenders’ risk and also enhance their levels of well-being (Yates et al., 2010), and the results from the present study, to a certain degree, supported the validity of the application of the GLM and SRM constructs to youth who sexually offended. What this means for clinical practice is that in addition to an assessment of static and dynamic risk factors for sexual recidivism, clinicians should also be encouraged to evaluate the youth’s self-regulation capacity and pathways associated with offending in order that these may also be differentially addressed in treatment (Yates & Ward, 2008). Ideally, the individualized treatment plan for the youth should contain (a) specific treatment targets, (b) strategies to address dynamic risk factors for sexual recidivism and motivations for offending, as well as (c) explicit good lives components (including strengths and positive experiences) to assist the youth to attain goods, which she or he values in life, via nonoffending ways (Yates & Ward, 2008).

### Limitations and Future Research

First, although the sample size of the present study is larger than most of the other empirical studies on GLM and SRM, it is still relatively small. Second, we relied on archival file data, instead of specific questions relating to GLM to code the primary human goods; hence, there was most likely an underestimation of the presence and function of primary human goods in youth sexual offending. Likewise, the offense pathways were coded from file records, and therefore, the retrospective methodology might have restricted the amount of information available compared with what that might have been supplied if interviews have been conducted. Another limitation pertained to the fact that we have not used a validated measure of the primary human goods, but a simple list of primary human goods that merely involved indicating the presence or absence of primary human goods that were linked to the sexual offending behavior in accordance with Ward and Gannon’s (2006) definitions. In spite of the CFPB psychologists and raters being formally trained in the GLM, the present study did not ascertain how reliable the psychological assessments and reports were in terms of documenting the evidence of primary human goods. Last, we had used charge sheets and statement of facts to classify the youth; these official records may not represent the full extent of the youth offending behavior.

Future research should use standardized interview and rating guides (e.g., Yates, Kingston, & Ward, 2009), and also provide details about the specific flaws that the youth exhibited in their bid to seek the primary human goods. In addition, prospective and repeated measures designs should be used whereby the primary human goods linked to sexual offending and offense pathways are assessed for changes over time (especially for those who have reoffended). Moreover, it is beneficial to examine the primary human goods and offense pathways in female youth who sexually offended, as there is currently little information on this population.

## Notes

1. A child victim is typically defined as a victim who is less than 12 years of age (e.g., Clift, Rajlic, & Gretton, 2009; Fanniff & Kolko, 2012; Rice, Harris, Lang, & Chaplin, 2012; Seto, Murphy, Page, & Ennis, 2003). In some studies (e.g., Aebi, Vogt, Plattner, Steinhausen, & Bessler, 2012; Hart-Kerkhoffs, Doreleijers, Jansen, van Wijk, & Bullens, 2009; Worling & Curwen, 2000), the victim must also be at least 3 or 4 years younger than the sexual offender in addition to the previous criterion.

2. All youth charged with sexual offenses in Singapore are assessed by Probation Officers for presentencing purposes, as well as possibly Child Protection Officers for care and protection purposes if it involves intrafamilial child victims. Notably, the Probation and Child Protection Services have systematically referred all their youth clients who had sexually offended to Clinical and Forensic Psychology Branch (CFPB) for psychological assessments.

3. The denominator is 164 because four youth could not be classified into one of the four pathways on the basis of their Avoidant/Approach and Passive/Active scores.

## References

[bibr1-1079063213499188] Aebi M. Vogt G. Plattner B. Steinhausen H.-C. Bessler C. (2012). Offender types and criminality dimensions in male juveniles convicted of sexual offenses. Sexual Abuse: A Journal of Research and Treatment, 24, 265-288. 21965467 10.1177/1079063211420449

[bibr2-1079063213499188] Arnett J. (1999). Adolescent storm and stress, reconsidered. American Psychologist, 54, 317-326. 10354802 10.1037//0003-066x.54.5.317

[bibr3-1079063213499188] Barnett G. Wood J. L. (2008). Agency, relatedness, inner peace, and problem solving in sexual offending: How sexual offenders prioritize and operationalized their good lives conceptions. Sexual Abuse: A Journal of Research and Treatment, 20, 444-467. 18948428 10.1177/1079063208325202

[bibr4-1079063213499188] Becker J. V. Kaplan M. S. (1988). The assessment of adolescent sexual offenders. Advances in Behavioral Assessment of Children and Families, 4, 97-118.

[bibr5-1079063213499188] Bhugra D. Popelyuk D. McMullen I. (2010). Paraphilias across cultures: Contexts and controversies. Journal of Sex Research, 47, 242-256. 20358463 10.1080/00224491003699833

[bibr6-1079063213499188] Bickley J. A. Beech A. R. (2002). An investigation of the Ward and Hudson Pathways Model of sexual offense process with child abusers. Journal of Interpersonal Violence, 17, 371-393.

[bibr7-1079063213499188] Chu C. M. Ng K. Fong J. Teoh J. (2012). Youth who sexually offended: The predictive validity of the ERASOR, J-SOAP-II, and YLS/CMI in a non-Western context. Sexual Abuse: A Journal of Research and Treatment, 24, 153-174. 21825111 10.1177/1079063211404250 PMC4449365

[bibr8-1079063213499188] Chu C. M. Thomas S. D. M. (2010). Adolescent sexual offenders: The relationship between typology and recidivism. Sexual Abuse: A Journal of Research and Treatment, 22, 218-233. 20458125 10.1177/1079063210369011 PMC4512030

[bibr9-1079063213499188] Cicchetti D. V. (1994). Guidelines, criteria, and rules of thumb for evaluating normed and standardized assessment instruments in psychology. Psychological Assessment, 6, 284-290.

[bibr10-1079063213499188] Clift R. J. W. Rajlic G. Gretton H. M. (2009). Discriminative and predictive validity of the penile plethysmography in adolescent sex offenders. Sexual Abuse: A Journal of Research and Treatment, 21, 335-362. 19587382 10.1177/1079063209338491

[bibr11-1079063213499188] Cochran W. Tesser A. (1996). The “what the hell” effect: Some effects of goal proximity and goal framing on performance. In Martin L. L. Tesser A. (Eds.), Striving and feeling: Interactions among goals, affect, and self-regulation (pp. 99-120). Hillsdale, NJ: Lawrence Erlbaum.

[bibr12-1079063213499188] Criminal Procedure Code, 68 Singapore Statutes (2012).

[bibr13-1079063213499188] Eaton D. Kann L. Kinchen S. Shanklin S. Ross J. Hawkins J., Centers for Disease Control and Prevention (CDC). (2008). Youth risk behavior surveillance—United States, 2007. Morbidity and Mortality Weekly Report Surveillance Summaries, 57, 1-131. 18528314

[bibr14-1079063213499188] Emmons R. A. (1999). The psychology of ultimate concerns. New York, NY: Guilford.

[bibr15-1079063213499188] Erikson E. (1968). Identity: Youth and crisis. New York, NY: Norton.

[bibr16-1079063213499188] Fanniff A. M. Kolko D. J. (2012). Victim age-based subtypes of juveniles adjudicated for sexual offenses: Comparisons across domains in an outpatient sample. Sexual Abuse: A Journal of Research and Treatment, 24, 224-264. 22127543 10.1177/1079063211416516

[bibr17-1079063213499188] Finkelhor D. (1984). Child sexual abuse: New theory and research. New York, NY: Free Press.

[bibr18-1079063213499188] Ford H. J. Rose J. Thrift S. (2009). An evaluation of the applicability of the self-regulation model to sexual offenders with intellectual disabilities. Journal of Forensic Psychiatry & Psychology, 20, 440-457.

[bibr19-1079063213499188] Gunby C. Woodhams J. (2010). Sexually deviant juveniles: Comparisons between the offender and offense characteristics of “child abusers” and “peer abusers.” Psychology, Crime & Law, 16, 47-64.

[bibr20-1079063213499188] Hall G. C. N. Hirschman R. (1991). Toward a theory of sexual aggression: A quadripartite model. Journal of Consulting and Clinical Psychology, 59, 662-669. 1955601 10.1037//0022-006x.59.5.662

[bibr21-1079063213499188] Hanson R. K. Morton-Bourgon K. E. (2005). The characteristics of persistent sexual offenders: A meta-analysis of recidivism studies. Journal of Consulting and Clinical Psychology, 73, 1154-1163. 16392988 10.1037/0022-006X.73.6.1154

[bibr22-1079063213499188] Hart-Kerkhoffs L. A. Doreleijers T. A. H. Jansen L. M. C. van Wijk A. P. H. Bullens R. A. R. (2009). Offense related characteristics and psychosexual development of juvenile sex offenders. Child and Adolescent Psychiatry and Mental Health, 3, 19. 19594889 10.1186/1753-2000-3-19 PMC2720916

[bibr23-1079063213499188] Hendriks J. Bijleveld C. C. J. H. (2004). Juvenile sexual delinquents: Contrasting child abusers with peer abusers. Criminal Behaviour and Mental Health, 14, 238-250. 15614327 10.1002/cbm.591

[bibr24-1079063213499188] Keeling J. A. Rose J. L. Beech A. R. (2006). A comparison of the application of the self-regulation model of the relapse process for mainstream and special needs sexual offenders. Sexual Abuse: A Journal of Research and Treatment, 18, 373-382. 17136624 10.1177/107906320601800405

[bibr25-1079063213499188] Kemper T. S. Kistner J. A. (2007). Offense history and recidivism in three victim-age-based groups of juvenile sex offenders. Sexual Abuse: A Journal of Research and Treatment, 19, 409-424. 17952596 10.1177/107906320701900406

[bibr26-1079063213499188] Kingston D. A. Yates P. M. Firestone P. (2012). The self-regulation model of sexual offending: Relationship to risk and need. Law and Human Behavior, 36, 215-224. 22667811 10.1037/h0093960

[bibr27-1079063213499188] Kingston D. A. Yates P. M. Simons D. Tyler C. (2009, 10). Self-regulation model of sexual offender treatment: Relationship to risk and the good lives model. Paper presented at the 28th Annual Convention of the Association for the Treatment of Sexual Abusers (ATSA), Dallas, TX.

[bibr28-1079063213499188] Kuo B. C. (2011). Culture’s consequences on coping: Theories, evidences, and dimensionalities. Journal of Cross-Cultural Psychology, 42, 1084-1100.

[bibr29-1079063213499188] Langdon P. E. Maxted H. Murphy G. H., & the Sex Offenders Treatment Services Collaborative-Intellectual Disabilities Group (SOTSEC-ID). (2007). An exploratory evaluation of the Ward and Hudson offending pathways model with sex offenders who have intellectual disabilities. Journal of Intellectual and Developmental Disability, 32, 94-105. 17613680 10.1080/13668250701364686

[bibr30-1079063213499188] Laws D. R. Ward T. (2011). Desistance and sexual offending: Alternatives to throwing away the keys. New York, NY: Guilford.

[bibr31-1079063213499188] Marshall W. L. Barbaree H. E. (1990). An integrated theory of the etiology of sexual offending. In Marshall W. L. Laws D. R. Barbaree H. E. (Eds.), Handbook of sexual assault: Issues, theories, and treatment of the offender (pp. 257-275). New York, NY: Plenum Press.

[bibr32-1079063213499188] Nisbet I. A. Wilson P. H. Smallbone S. W. (2004). A prospective longitudinal study of sexual recidivism among adolescent sex offenders. Sexual Abuse: A Journal of Research and Treatment, 16, 223-234. 15326882 10.1177/107906320401600304

[bibr33-1079063213499188] Penal Code, 224 Singapore Statutes, (2008).

[bibr34-1079063213499188] Rice M. E. Harris G. T. Lang C. Chaplin T. C. (2012). Adolescent who sexually offended: Is phallometry valid? Sexual Abuse: A Journal for Research and Treatment, 24, 133-152. 10.1177/1079063211404249 21960516

[bibr35-1079063213499188] Seto M. C. Lalumière M. L. (2010). What is so special about male adolescent sexual offending? A review and test of explanations through meta-analyses. Psychological Bulletin, 136, 526-575. 20565168 10.1037/a0019700

[bibr36-1079063213499188] Seto M. C. Murphy W. D. Page J. Ennis L. (2003). Detecting anomalous sexual interests among juvenile sex offenders. Annals of the New York Academy of Sciences, 989, 118-130. 12839891 10.1111/j.1749-6632.2003.tb07298.x

[bibr37-1079063213499188] Singapore Police Force. (2012). Crime situation for 2011. Retrieved from http://www.spf.gov.sg/stats/stats2011_intro.htm

[bibr38-1079063213499188] Steinberg L. Albert D. Cauffman E. Banich M. Graham S. Woolard J. (2008). Age differences in sensation seeking and impulsivity as indexed by behavior and self-report: Evidence for a dual systems model. Developmental Psychology, 44, 1764-1778. 18999337 10.1037/a0012955

[bibr39-1079063213499188] Viljoen J. L. Mordell S. Beneteau J. L. (2012). Prediction of adolescent sexual reoffending: A meta-analysis of the J-SOAP-II, ERASOR, J-SORRAT-II, and Static-99. Law and Human Behavior, 36, 423-438. 22353046 10.1037/h0093938

[bibr40-1079063213499188] Vizard E. Hickey N. French L. McCrory E. (2007). Children and adolescents who present with sexually abusive behaviour: A UK descriptive study. Journal of Forensic Psychiatry & Psychology, 18, 59-73.

[bibr41-1079063213499188] Ward T. (2002). Good lives and the rehabilitation of offenders: Promises and problems. Aggression and Violent Behavior, 7, 513-528.

[bibr42-1079063213499188] Ward T. Gannon T. A. (2006). Rehabilitation, etiology, and self-regulation: The comprehensive good lives model of treatment for sexual offenders. Aggression and Violent Behavior, 11, 77-94.

[bibr43-1079063213499188] Ward T. Hudson S. M. (1998). A model of relapse prevention in sexual offenders. Journal of Interpersonal Violence, 13, 700-725.

[bibr44-1079063213499188] Ward T. Mann R. (2004). Good lives and the rehabilitation of offenders: A positive approach to treatment. In Linley A. Joseph S. (Eds.), Positive psychology in practice (pp. 598-616). New York, NY: John Wiley.

[bibr45-1079063213499188] Ward T. Maruna S. (2007). Rehabilitation: Beyond the risk assessment paradigm. London, England: Routledge.

[bibr46-1079063213499188] Ward T. Siegert R. J. (2002). Toward a comprehensive theory of child sexual abuse: A theory knitting perspective. Psychology, Crime, & Law, 9, 197-248.

[bibr47-1079063213499188] Ward T. Stewart C. A. (2003). The treatment of sex offenders: Risk management and good lives. Professional Psychology: Research and Practice, 34, 353-360.

[bibr48-1079063213499188] Ward T. Yates P. M. Willis G. M. (2012). The good lives model and the risk need responsivity model: A critical response to Andrews, Bonta, and Wormith (2011). Criminal Justice and Behavior, 39, 94-110.

[bibr49-1079063213499188] Webster S. D. (2005). Pathways to sexual offense recidivism following treatment: An examination of the Ward and Hudson self-regulation model of relapse. Journal of Interpersonal Violence, 20, 1175-1196. 16162485 10.1177/0886260505278532

[bibr50-1079063213499188] Willis G. M. Yates P. M. Gannon T. A. Ward T. (2013). How to integrate the Good Lives Model into treatment programs for sexual offending: An introduction and overview. Sexual Abuse: A Journal for Research and Treatment, 25, 123-142. 10.1177/1079063212452618 22798205

[bibr51-1079063213499188] Worling J. R. Bookalam D. Litteljohn A. (2012). Prospective validity of the Estimate of Risk of Adolescent Sexual Offense Recidivism (ERASOR). Sexual Abuse: A Journal for Research and Treatment, 24, 203-223. 10.1177/1079063211407080 21969313

[bibr52-1079063213499188] Worling J. R. Curwen T. (2000). Adolescent sexual offender recidivism. Success of specialized treatment and implications for risk prediction. Child Abuse & Neglect, 24, 965-982. 10905420 10.1016/s0145-2134(00)00147-2

[bibr53-1079063213499188] Worling J. R. Curwen T. (2001). Estimate of risk of adolescent sexual offense recidivism (Version 2.0). Toronto, Ontario, Canada: Ontario Ministry of Community and Social Services.

[bibr54-1079063213499188] Yates P. M. Kingston D. (2006). The self-regulation model of sexual offending: The relationship between offence pathways and static and dynamic sexual offence risk. Sexual Abuse: A Journal of Research and Treatment, 18, 259-270. 16871449 10.1177/107906320601800304

[bibr55-1079063213499188] Yates P. M. Kingston D. A. Simons D. A. Tyler C. (2009, 10). The good lives model of rehabilitation applied to treatment: Assessment and relationship to treatment progress and compliance. Paper presented at the 28th Annual Convention of the Association for the Treatment of Sexual Abusers (ATSA), Dallas, TX.

[bibr56-1079063213499188] Yates P. M. Kingston D. A. Ward T. (2009). The self-regulation model of the offense and relapse process: Volume 3: A guide to assessment and treatment planning using the integrated Good Lives/Self-Regulation Model of sexual offending. Victoria, British Columbia, Canada: Pacific Psychological Assessment Corporation.

[bibr57-1079063213499188] Yates P. M. Prescott D. Ward T. (2010). Applying the good lives and self-regulation models to sex offender treatment: A practical guide for clinicians. Brandon, VT: Safer Society Press.

[bibr58-1079063213499188] Yates P. M. Ward T. (2008). Good lives, self-regulation, and risk management: An integrated model of sexual offender assessment and treatment. Sexual Abuse in Australia and New Zealand: An Interdisciplinary Journal, 1, 3-20.

